# Field evaluations of four SARS-CoV-2 rapid antigen tests during SARS-CoV-2 Delta variant wave in South Africa

**DOI:** 10.1186/s41512-023-00151-3

**Published:** 2023-07-25

**Authors:** Natasha Samsunder, Gila Lustig, Slindile Ngubane, Thando Glory Maseko, Santhuri Rambaran, Sinaye Ngcapu, Stanley Nzuzo Magini, Lara Lewis, Cherie Cawood, Ayesha B. M. Kharsany, Quarraisha Abdool Karim, Salim Abdool Karim, Kogieleum Naidoo, Aida Sivro

**Affiliations:** 1 Centre for the AIDS Programme of Research in South Africa (CAPRISA), Durban, South Africa; 2 Department of Medical Microbiology, University of KwaZulu-Natal, Durban, South Africa; 3 Epicentre AIDS Risk Management (Pty) Limited, Durban, South Africa; 4 Department of Epidemiology, Columbia University, New York City, USA; 5 South African Medical Research Council (SAMRC), CAPRISA-TB-HIV Pathogenesis and Treatment Research Unit, University of KwaZulu-Natal Nelson R. Mandela School of Medicine, Durban, South Africa; 6 JC Wilt Infectious Disease Research Centre, National Microbiology Laboratory, Public Health Agency of Canada, Winnipeg, MB Canada; 7 Department of Medical Microbiology and Infectious Diseases, University of Manitoba, Winnipeg, MB Canada

**Keywords:** COVID-19, SARS-CoV-2, Antigen rapid diagnostic test, Performance evaluation

## Abstract

**Background:**

Rapid antigen tests detecting SARS-CoV-2 were shown to be a useful tool in managing the COVID-19 pandemic. Here, we report on the results of a prospective diagnostic accuracy study of four SARS-CoV-2 rapid antigen tests in a South African setting.

**Methods:**

Rapid antigen test evaluations were performed through drive-through testing centres in Durban, South Africa, from July to December 2021. Two evaluation studies were performed: nasal Panbio COVID-19 Ag Rapid Test Device (Abbott) was evaluated in parallel with the nasopharyngeal Espline SARS-CoV-2 Ag test (Fujirebio), followed by the evaluation of nasal RightSign COVID-19 Antigen Rapid test Cassette (Hangzhou Biotest Biotech) in parallel with the nasopharyngeal STANDARD Q COVID-19 Ag test (SD Biosensor). The Abbott RealTime SARS-CoV-2 assay was used as a reference test.

**Results:**

Evaluation of Panbio and Espline Ag tests was performed on 494 samples (31% positivity), while the evaluation of Standard Q and RightTest Ag tests was performed on 539 samples (13.17% positivity). The overall sensitivity for all four tests ranged between 60 and 72% with excellent specificity values (> 98%). Sensitivity increased to > 80% in all tests in samples with cycle number value < 20. All four tests performed best in samples from patients presenting within the first week of symptom onset.

**Conclusions:**

All four evaluated tests detected a majority of the cases within the first week of symptom onset with high viral load.

**Supplementary Information:**

The online version contains supplementary material available at 10.1186/s41512-023-00151-3.

## Introduction

Diagnostic testing has proven to be imperative for the management of SARS-CoV-2/COVID-19 in the context of reducing transmission and outbreak control [1]. While the gold standard RT-PCR test is highly sensitive and specific, there are several disadvantages including cost, complexity and length of the process, and need for specialised equipment and trained personal. The use and availability of rapid antigen tests (RDTs) for the diagnosis of SARS-CoV-2 infection have significantly increased over the last year of the pandemic. RDTs are affordable, fast (10–30 min), simple, and do not require specialised laboratory facilities or highly trained personal. Although their sensitivity is lower compared to a laboratory-based RT-PCR, antigen-based RDTs can detect infection early following symptom onset when the viral load is high, thereby offering quick screening and detection of SARS-CoV-2/COVID-19 among high-risk groups [2].

As of May 2022, South African Health Products Regulatory Authority (SAHPRA) has approved 53 RDT test for use in South Africa [3]. While the World Health Organization (WHO) recommends a minimum of 80% sensitivity and 97% specificity for rapid antigen diagnostics tests to be approved, there is significant variation in RDT performance depending on the study settings [4–10] As an example, the reported performance of commonly used Standard Q COVID-19 Ag test varies between 28.7 [11] and 89.2% [12] depending on the prevalence and patient group. Furthermore, there continues to be a limited number of reports on RDT field performance in low- and middle-income country (LMIC) settings. Here, we evaluate the performance of four rapid antigen tests in comparison with the Abbott RT-PCR assay during the B.1.617.2 Delta variant wave in KwaZulu-Natal, South Africa.

## Methods

### Study participant recruitment

Rapid antigen test evaluations were performed between July and December 2021 through drive-through testing centres in Durban, South Africa. Adult participants (age =  > 18) meeting any of the following criteria were enrolled in the study: tested COVID-19 positive in the previous 7 days, the presence of COVID-19 symptoms in the previous seven days, exposed to COVID-19 5–10 days ago, healthcare worker, or doctor referral for testing. Drive-through testing centres were freely accessible with no referral necessary for testing. Data on screened out individuals that did not fit the study enrolment criteria is not available. Two separate evaluation studies were performed: first, the Espline SARS-CoV-2 Ag test [Fujirebio, nasopharyngeal (NP)] [13] was evaluated in parallel with the Panbio COVID-19 Ag Rapid Test Device (Abbott, nasal) [14]; this was followed by the evaluation of RightSign COVID-19 Antigen Rapid test Cassette (Hangzhou Biotest Biotech, nasal) [15] in parallel with the STANDARD Q COVID-19 Ag test (SD Biosensor, NP) [16] on a different group of participants. The Abbott RealTime SARS-CoV-2 assay (target sequences in the SARS-CoV-2 RdRp and N genes of the SARS-CoV-2 genome) [17] was used as a reference test. The Abbott RealTime SARS-CoV-2-positive results are reported with cycle number (CN) values that are equivalent to cycle threshold values more commonly used by other assays [18]. Following informed consent, participants filled in a questionnaire on basic demographic and clinical data and provided samples for the study. Study participants were provided with the results of the South African Health Products Regulatory Authority (SAHPRA) and approved rapid antigen test on site, and Abbott RT-PCR results were reported within 24 h of sample collection. At the time of the study, the nasal Panbio Ag test and NP STANDARD Q Ag test were SAHPRA approved. The study protocol was written before recruitment began. The protocol was not published. Ethics for the parent study was obtained on 24 March 2020. The evaluated tests were selected and provided by the Foundation for Innovative New Diagnostics (FIND) based on the availability and evaluation needs at the time of the study. Minimum of 50 positive cases for each test was required for evaluation. Test evaluations were continued until study ran out of available kits.

### Sample collection and processing

Study participants provided three swabs: one nasal and two nasopharyngeal. Nasal swab was collected first in order to avoid cross-contamination between sites, followed by a NP swab for second rapid antigen test, and followed by the NP swab for SARS-CoV-2 RT-PCR. Swabs were collected, and rapid antigen tests were performed and interpreted by trained medical staff on site. The results of the tests were interpreted and recorded by two staff members independently. Swabs for RT-PCR were sent to the central laboratory at room temperature without additives. Swab was resuspended in 2 ml of viral transport media (VTM) and processed within 3 h of sample collection. All assays were performed as per manufacturer’s protocol.

### Statistical analysis

Statistical analysis was performed using SPSS version 27 and GraphPad Prism version 8.3.1 (GraphPad software, La Jolla, CA, USA). Study analysis was prespecified by FIND. Test performance characteristics [sensitivity, specificity, positive predictive value (PPV), and negative predictive value (NPV)] were calculated in reference to Abbott RealTime SARS-CoV-2 assay results. Wilson’s score method was used to calculate the 95% confidence intervals to assess the level of uncertainty induced by sample size. Test performance was assessed across different categories including the presence and duration of symptoms (no symptoms, symptoms =  > 7 days, and symptoms < 7 days) and CN values (< 20 and < 25) as indicator of viral load and infectiousness [19]. In case of missing data, a complete-case analysis approach was used. The measure of agreement between the assays was evaluated using Cohen’s kappa coefficient [20]. A *t*-test was used to assess differences in CN values between true-positive and false-negative results.

## Results

### Study sample characteristics

The evaluation of nasal Panbio and NP Espline Ag tests was done on 494 samples (Table 1) between 21st of July and 19th of August 2021. The median age of study participants was 34 [interquartile range (IQR) 24–47] with 57.29% of participants being female. The SARS-CoV-2 positivity was 31.00% with median CN value of 9.06 (*IQR* 5.90–16.90), with all positive samples having a *CN* < 31. Majority of the study participants (57.36%) presented for testing during the first week post-symptom onset.

**Table 1:** Study participant/sample characteristics

	Evaluation 1	Evaluation 2
**Sample size (*N*)**	494	539
**Age, years (median, IQR)**	34, 24–47	36, 24–50
**Gender (%, n/N female)**	57.29, 283/494	51.76, 279/539
**% positivity (n/N)**	31.00, 153/494	13.17 (71/539)
**Presence of symptoms**		
Asymptomatic/presymptomatic (%, n/N)	33.33, 129/387	29.50, 159/539
< 7-day PSO (%, n/N)	57.36, 222/387	64.01, 345/539
= > 7-day PSO (%, n/N)	9.30, 36/387	6.49, 35/539
**HIV positive (%, n/N)#**	0.42, 2/481	0.20, 1/539
**CN value (median, IQR)**	9.06, 5.90–16.90	15.08, 11.53–23.86
**Oxygen saturation (median, IQR)**	96, 95–98	96, 95–97

Missing data: evaluation 1 (HIV status, 13; oxygen saturation, 72; days of symptom onset, 107); evaluation 2 (oxygen saturation, 26). *PSO* post-symptom onset. #Self-reported

The evaluation of NP Standard Q and nasal RightSign Ag tests was done on 539 samples (Table 1) collected between 15th of September and 8th of December 2021. The median age of study participants was 36 (*IQR* 24–50) with 51.76% being female. The SARS-CoV-2 positivity was 13.17% with a median CN value of 15.08 (*IQR* 11.53–23.86), with all positive samples having a CN value < 31. Majority of the study participants presented for testing during first week post-symptom onset (64.01%).

### Test performance evaluation

**Fig. 1:**
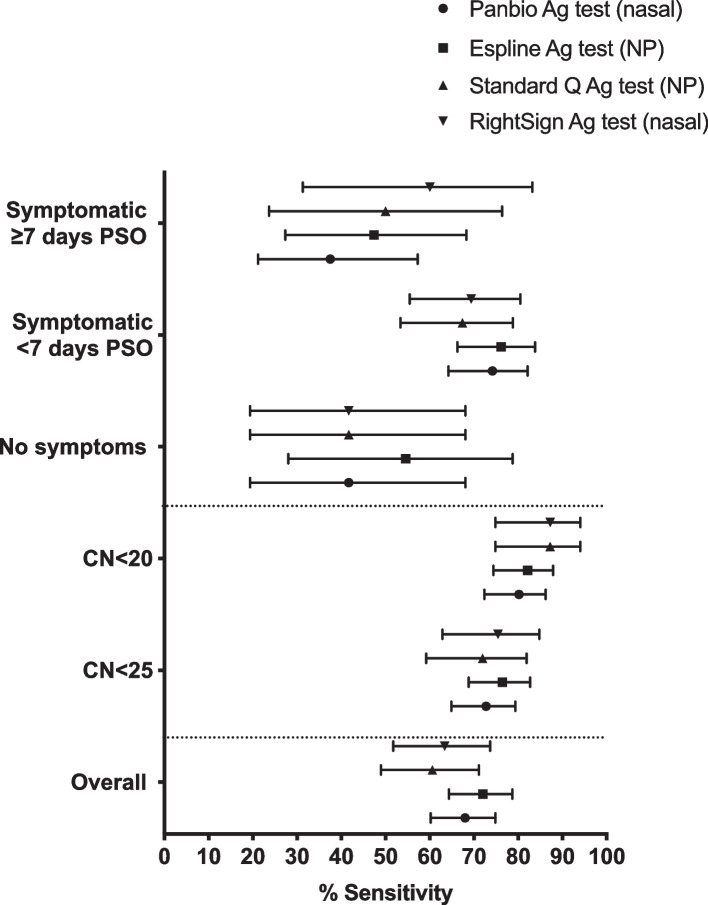
Sensitivity for the Panbio COVID-19 Ag Rapid Test Device (nasal), Espline SARS-CoV-2 Ag test (NP), STANDARD Q COVID-19 Ag test (NP), and RightSign COVID-19 Antigen Rapid test Cassette (nasal) across different sample categories: SARS-CoV-2 CN value and days post-symptom onset. Error bars represent 95% confidence intervals. PSO, post-symptom onset

The overall performance of nasal Panbio and NP Espline Ag tests is summarised in Sup. Table 1 and Fig. 1. The overall sensitivity and specificity of nasal Panbio Ag test were 67.97% (95% *CI* 60.22–74.85) and 98.53% (95% *CI* 96.61–99.37), respectively. The overall performance of NP Espline Ag tests was slightly higher with overall sensitivity and specificity of 72.00% (95% *CI* 64.33–78.67) and 99.71% (95% *CI* 98.35–99.95), respectively. The sensitivity of both tests increased in samples with lower CN values (increased viral load): for samples with *CN* < 20 sensitivity of nasal Panbio Ag, test was 80.16% (95% *CI* 72.35–86.18), and sensitivity of NP Espline Ag test was 82.11% (95% *CI* 74.40–87.88). Both tests performed best in samples from individuals presenting during the first week of symptom onset, with sensitivities of 74.16% (95% *CI* 64.20–82.12) for Panbio and 76.14% (95% *CI* 66.26–83.83) for Espline Ag tests, and worst in individuals presenting more than 7-day post-symptom onset, with sensitivities of 37.50% (95% *CI* 21.16–57.29) for Panbio and 47.37% (95% *CI* 27.33–68.29) for Espline Ag test.

The performance of NP Standard Q and nasal RightSign Ag test is summarised in Sup. Table 2 and Fig. 1. The overall sensitivity and specificity of NP Standard *Q*-test were 60.56% (95% *CI* 48.94–71.11) and 99.79% (95% *CI* 98.80–99.96), respectively. The overall sensitivity of nasal RightSign Ag test was 63.38% (95% *CI* 51.76–73.63) with specificity of 100.00% (95% *CI* 99.19–100.00). The sensitivity of both tests increased in samples with lower CN values, being 87.23% (95% *CI* 74.83–94.02) for both Standard Q and RightSign Ag test in samples with *CN* < 20.

The kappa coefficient ranged between 0.72 and 0.78 indicating substantial agreement between the tested RDTs and the Abbott RT-PCR assay. As expected, majority of false-negative (FN) results for all 4 tests occurred in samples with higher CN values (Sup. Figure 1). There were 4 invalid test results with Espline Ag test (0.8%), with no invalid results for the remaining three tests.

## Discussion

In this study, we determined the sensitivity and specificity of four RDTs for the detection of SARS-CoV-2 virus in respiratory specimens during the Delta wave of infections in Durban, South Africa.

The sensitivity of the four evaluated tests ranged from 60.55 to 87.23% with high specificity (ranging from 83.33 to 100%). The observed sensitivity of the evaluated tests is comparable with previously published studies from different settings [2, 21]. As reported previously, we observed similar results between the nasal and nasopharyngeal assays performed on equivalent samples [22, 23]. The sensitivity of each of the RDTs increased in samples with lower CN values, increasing above 80% in samples with *CN* < 20. As previously reported, all four tests performed best in samples from individuals presenting within the first week of symptom onset when the SARS-CoV-2 viral load is highest [24–27]. While the rapid antigen tests have often been criticised for low sensitivity and high rate of false-negative results, the majority of the false-negative results are observed in samples with higher C threshold/number values (low viral load) that likely have a limited potential for fulling further viral transmission [2, 24, 28, 29]. In fact, antigen-based RDTs were shown to correlate better with replication-competent SARS-CoV-2 compared to RT-PCR [30] further supporting the use of rapid antigen tests in identifying individuals who are at high potential to transmit SARS-CoV-2. Additionally, low cost and scalability represent an important advantage over standard RT-PCR tests, especially in low- and middle-income countries.

There are several limitations to our study. We did not have access to participant vaccination status and the presence and timing of previous natural infections, and we therefore could not assess the impact of previous immunity on SARS-CoV-2 viral load and rapid antigen test performance. Even though viral sequencing was not available in our study, the period overlaps with the Delta wave of infections in KwaZulu-Natal [31]. Additionally, we have no data on patients that were screened out due to not meeting the study enrollment criteria. While none of the evaluated tests satisfied the WHO requirements for the > 80% sensitivity in the overall sample group, they are still a valuable tool in identifying infected individuals within the first week of symptom onset and those with high viral loads and could play an important role in limiting transmission and controlling the COVID-19 pandemic. Rapid antigen tests remain a useful tool for rapid screening for COVID-19 in congregate settings as well as for “test to work” strategies in order to reduce/slow down spread of the virus. This study provides valuable information of the performance of rapid antigen tests in drive-through centres in South Africa.

## Supplementary Information

**Additional file 1: Supplementary figures: Fig. 1.** Differences in SARS-CoV-2 CN values between true positive and false negative samples for A) Panbio COVID-19 Ag Rapid Test Device (nasal) B) Espline SARS-CoV-2 Ag test (NP) C) STANDARD Q COVID-19 Ag test (NP) D) RightSign COVID-19 Antigen Rapid test Cassette (nasal). **Supplementary tables: Table 1.** Estimations of test performance for Panbio COVID-19 Ag Rapid Test Device (nasal) and Espline SARS-CoV-2 Ag test (NP) with respect to RT-PCR CN values (A) and presence/duration of symptoms (B). *Represents overall sensitivity and specificity (CN values for all positive samples were <31). **Table 2.** Estimations of test performance for STANDARD Q COVID-19 Ag test (NP) and RightSign COVID-19 Antigen Rapid test Cassette (Nasal Swab) with respect to RT-PCR CN values (A) and presence/duration of symptoms (B). *Represents overall sensitivity and specificity (CN values for all positive samples were <31).

## Data Availability

De-identified patient-level data can be accessed by contacting the corresponding author with a detailed description of the research question.
