# Supplementary material for: Field evaluations of four SARS-CoV-2 rapid antigen tests during SARS-CoV-2 Delta variant wave in South Africa
Source: Diagn Progn Res. 2023 Jul 25;7:14. doi: 10.1186/s41512-023-00151-3 (PMC10369830; doi:10.1186/s41512-023-00151-3)
Supplement: Supplementary file 1 — Additional file 1: Supplementary figures: Fig. 1. Differences in SARS-CoV-2 CN values between true positive and false negative samples for A) Panbio COVID-19 Ag Rapid Test Device (nasal) B) Espline SARS-CoV-2 Ag test (NP) C) STANDARD Q COVID-19 Ag test (NP) D) RightSign COVID-19 Antigen Rapid test Cassette (nasal). Supplementary tables: Table 1. Estimations of test performance for Panbio COVID-19 Ag Rapid Test Device (nasal) and Espline SARS-CoV-2 Ag test (NP) with respect to RT-PCR CN values (A) and presence/duration of symptoms (B). *Represents overall sensitivity and specificity (CN values for all positive samples were <31). Table 2. Estimations of test performance for STANDARD Q COVID-19 Ag test (NP) and RightSign COVID-19 Antigen Rapid test Cassette (Nasal Swab) with respect to RT-PCR CN values (A) and presence/duration of symptoms (B). *Represents overall sensitivity and specificity (CN values for all positive samples were <31). [file 41512_2023_151_MOESM1_ESM.docx]

**Supplementary material:**

**Sup. Figure 1.** Differences in SARS-CoV-2 CN values between true positive and false negative samples for **A**) Panbio COVID-19 Ag Rapid Test Device (nasal) **B**) Espline SARS-CoV-2 Ag test (NP) **C)** STANDARD Q COVID-19 Ag test (NP) **D)** RightSign COVID-19 Antigen Rapid test Cassette (nasal).

**Sup. Table 1. Estimations of test performance for Panbio COVID-19 Ag Rapid Test Device (nasal) and Espline SARS-CoV-2 Ag test (NP) with respect to RT-PCR CN values (A) and presence/duration of symptoms (B)**

|  | Panbio COVID-19 Ag Rapid Test Device (nasal) | | | Espline SARS-CoV-2 Ag test (NP) | | |
| --- | --- | --- | --- | --- | --- | --- |
| 1. **Ct category** | **CN<31*** | **CN<25** | **CN<20** | **CN<31*** | **CN<25** | **CN<20** |
| **True positive** | 104 | 104 | 101 | 108 | 107 | 101 |
| **False positive** | 5 | - | - | 1 | - | - |
| **True negative** | 336 | - | - | 339 | - | - |
| **False negative** | 49 | 39 | 25 | 42 | 33 | 22 |
|  | | | | | | |
| **Sensitivity (%, 95% CI)** | 67.97 (60.22-74.85) | 72.73 (64.90-79.36) | 80.16 (72.35-86.18) | 72.00 (64.33-78.67) | 76.43 (68.75-82.69) | 82.11 (74.40-87.88) |
| **Specificity (%, 95% CI)** | 98.53 (96.61-99.37) | - | - | 99.71 (98.35-99.95) | - | - |
| **PPV (%, 95% CI)** | 95.41 (89.71-98.02) | - | - | 99.08 (94.99-99.84 | - | - |
| **NPV (%, 95% CI)** | 87.27 (83.57-90.24) | - | - | 88.98 (85.43-91.74) | - | - |
| **Invalid (%, n/N)** | 0 | - | - | 0.8%,4/494 | - | - |
| **Kappa (%, 95% CI)** | 0.72 (0.65-0.79) | - | - | 0.78 (0.71-0.84) | - | - |
|  |  |  |  |  |  |  |
| 1. **Symptom category** | **Asymptomatic** | **Symptomatic**  **<7 days** | **Symptomatic**  **>=7 days** | **Asymptomatic** | **Symptomatic**  **<7 days** | **Symptomatic**  **>=7 days** |
| **True positive** | 5 | 66 | 9 | 6 | 67 | 9 |
| **False positive** | 0 | 3 | 2 | 0 | 1 | 0 |
| **True negative** | 117 | 130 | 15 | 117 | 131 | 17 |
| **False negative** | 7 | 23 | 10 | 5 | 21 | 10 |
|  |  |  |  |  |  |  |
| **Sensitivity (%, 95% CI)** | 41.67 (19.33-68.05) | 74.16 (64.20-82.12) | 37.50 (21.16-57.29) | 54.55 (28.01-78.73) | 76.14 (66.26-83.83) | 47.37 (27.33-68.29) |
| **Specificity (%, 95% CI)** | 100.00 (96.82-100.00) | 97.74 (93.58-99.23) | 83.33 (55.20-95.30) | 100.00 (96.82-100.00) | 99.24 (95.83-99.87) | 100.00 (81.57-100.00) |
| **PPV (%, 95% CI)** | 100.00 (56.55-100.00) | 95.65 (87.98-98.51) | 81.82 (52.30-94.86) | 100.00 (60.97-100.00) | 98.53 (92.13-99.74) | 100.00 (70.09-100.00) |
| **NPV (%, 95% CI)** | 94.35 (88.81-97.24) | 84.97 (78.45-89.77) | 40.00 (23.40-59.26) | 95.90 (90.76-98.24) | 86.18 (79.80-90.78) | 62.96 (44.23-78.47) |

*Represents overall sensitivity and specificity (CN values for all positive samples were <31)

**Sup. Table 2. Estimations of test performance for STANDARD Q COVID-19 Ag test (NP) and RightSign COVID-19 Antigen Rapid test Cassette (Nasal Swab) with respect to RT-PCR CN values (A) and presence/duration of symptoms (B)**

|  | STANDARD Q COVID-19 Ag test (NP) | | | RightSign COVID-19 Antigen Rapid test Cassette (nasal) | | |
| --- | --- | --- | --- | --- | --- | --- |
| 1. **Ct category** | **CN<31*** | **CN<25** | **CN<20** | **CN<31*** | **CN<25** | **CN<20** |
| **True positive** | 43 | 41 | 41 | 45 | 43 | 41 |
| **False positive** | 1 | - | - | 0 | - | - |
| **True negative** | 467 | - | - | 468 | - | - |
| **False negative** | 28 | 16 | 6 | 26 | 14 | 6 |
|  | | | | | | |
| **Sensitivity (%, 95% CI)** | 60.56 (48.94-71.11) | 71.93 (59.17-81.92) | 87.23 (74.83-94.02) | 63.38 (51.76-73.63) | 75.44 (62.90-84.77) | 87.23 (74.83-94.02) |
| **Specificity (%, 95% CI)** | 99.79 (98.80-99.96) | - | - | 100.00 (99.19-100.00) | - | - |
| **PPV (%, 95% CI)** | 97.73 (88.19-99.60) | - | - | 100.00 (92.13-100.00) | - | - |
| **NPV (%, 95% CI)** | 94.34 (91.95-96.06) | - | - | 94.74 (92.40-96.38) | - | - |
| **Invalid (%, n/N)** | 0 | - | - | 0 | - | - |
| **Kappa (%, 95% CI)** | 0.72 (0.62-0.82) | - | - | 0.75 (0.66-0.84) | - | - |
|  |  |  |  |  |  |  |
| 1. **Symptom category** | Asymptomatic | Symptomatic  <7 days PSO | Symptomatic  >=7 days PSO | Asymptomatic | Symptomatic  <7 days PSO | Symptomatic  >=7 days PSO |
| **True positive** | 5 | 33 | 5 | 5 | 34 | 6 |
| **False positive** | 0 | 1 | 0 | 0 | 0 | 0 |
| **True negative** | 147 | 295 | 25 | 147 | 296 | 25 |
| **False negative** | 7 | 16 | 5 | 7 | 15 | 4 |
|  |  |  |  |  |  |  |
| **Sensitivity (%, 95% CI)** | 41.67 (19.33-68.05) | 67.35 (53.38-78.79) | 50.00 (23.66-76.34) | 41.67 (19.33-68.05) | 69.39 (55.47-80.48) | 60.00 (31.27-83.18) |
| **Specificity (%, 95% CI)** | 100.00 (97.45-100.00) | 99.66 (98.11-99.94) | 100.00 (86.68-100.00) | 100.00 (97.45-100.00) | 100.00 (98.72-100.00) | 100.00 (86.68-100.00) |
| **PPV (%, 95% CI)** | 100.00 (56.55-100.00) | 97.06 (85.08-99.48) | 100.00 (56.55-100.00) | 100.00 (56.55-100.00) | 100.00 (89.85-100.00) | 100.00 (60.97-100.00) |
| **NPV (%, 95% CI)** | 95.45 (90.92-97.78) | 94.86 (91.81-96.81) | 83.33(66.44-92.66) | 95.45 (90.92-97.78) | 95.18 (92.20-97.06) | 86.21 (69.44-94.50) |

*Represents overall sensitivity and specificity (CN values for all positive samples were <31)
